# Analysis of the Transcriptome of the Red Seaweed *Grateloupia imbricata* with Emphasis on Reproductive Potential

**DOI:** 10.3390/md16120490

**Published:** 2018-12-07

**Authors:** Pilar Garcia-Jimenez, Carlos Llorens, Francisco J. Roig, Rafael R. Robaina

**Affiliations:** 1 Departamento de Biología, Facultad de Ciencias del Mar, Universidad de Las Palmas de Gran Canaria, Las Palmas de Gran Canaria, E-35017 Las Palmas, Spain; rafael.robaina@ulpgc.es; 2 Biotechvana S.L., Vivero Parc Cientific Universitat de Valencia, Catedrático José Beltran 2, 46980 Paterna, Valencia, Spain; carlos.llorens@biotechvana.com (C.L.); francisco.roig@biotechvana.com (F.J.R.)

**Keywords:** carbon sources, growth regulators, red algae, reproduction, transcriptome shotgun assembly

## Abstract

*Grateloupia imbricata* is an intertidal marine seaweed and candidate model organism for both industry and academic research, owing to its ability to produce raw materials such as carrageenan. Here we report on the transcriptome of *G. imbricata* with the aim of providing new insights into the metabolic pathways and other functional pathways related to the reproduction of *Grateloupia* species. Next-generation sequencing was carried out with subsequent de novo assembly and annotation using state-of-the-art bioinformatic protocols. The results show the presence of transcripts required for the uptake of glycerol, which is a specific carbon source for in vitro culture of *G. imbricata* and nucleotide sequences that are involved in polyamine-based biosynthesis, polyamine degradation, and metabolism of jasmonates and ethylene. Polyamines, ethylene and methyl jasmonate are plant growth regulators that elicit the development and maturation of cystocarps and the release of spores from seaweeds. Our results will inform studies of the mechanisms that control polysaccharide accumulation, cystocarp formation and spore release. Moreover, our transcriptome information clarifies aspects of red seaweed carposporogenesis with potential benefits for enhancing reproduction.

## 1. Introduction

Next-generation sequencing has been used to obtain complete genome sequences of a species and transcriptome profiling. Altogether these two aspects allow to address the unknown aspects of algae physiology. In red seaweeds, sequencing approaches have been carried out on species of commercial value such as those of the genera *Pyropia*, *Gracilaria* and *Chondrus* [1,2], reporting aspects related to seaweed evolution and responses to different abiotic stresses. Despite the advancements made with these genera, other red seaweeds may also represent suitable models for studies of molecular and physiological processes, particularly considering the progress made on in vitro cultivation of individual species while varying the carbon source(s) and growth regulators. Maximization of the potential of seaweed culture to provide foodstuffs and exploit industry-valued products will depend on the genetic modification of seaweeds to enhance particular morphological features and growth rates and to delay fertility.

*Grateloupia imbricata* is a red algal taxon of major interest for the biotechnology industry because it produces raw materials such as carrageenan. *G. imbricata* is an intertidal macroalga inhabiting the rocky pools of the Canary Islands; it is fully exposed to light, evaporation and an increase of temperature during low tide. Reproduction in *G. imbricata* occurs by both sexual and asexual processes. Fertilization involves the fusion of a non-flagellated male gamete, the spermatium, with a sessile egg-containing cell, the carpogonium on the female gametangial thalli. After fertilization, the zygote is retained on the female gametangial thalli where it develops into the third generation, the carposporophyte. Carposporophyte of the red algae may bear a macroscopically visible reproductive structure, the cystocarp. Cystocarps appear as small dark spots (1–5 mm diameter) embedded in the thalli, and eventually release the carpospores which serve as the agents of recruitment for the tetrasporophyte generation, a tetraspore producing phase. Tetraspores originate from male and female gametangial thalli, completing the life cycle.

Culture of *G. imbricata* in glycerol-containing media can increase polymer biosynthesis [3,4], and this seaweed responds to growth regulators as elicitors of carposporogenesis and sporulation [4,5,6,7,8,9]. The focus of research with this seaweed species has since transitioned from growth performance to the identification of gene-encoding proteins responsible for the synthesis of polymers and the small molecules ethylene, methyl jasmonate, and polyamines as the main elicitors of cystocarp maturation and spore release [10,11,12]. Owing to the relative paucity of gene information for red seaweeds, we hypothesized that improving the functional annotation of seaweed genes would provide insight into gene mechanisms in particular and seaweed biology in general. Hence, we acquired data using sequencing technologies to assemble and annotate the transcriptome of *G. imbricata* toward the goal of establishing a global functional profile for this species with particular focus on transcripts related to the synthesis of biopolymers, growth, development, and reproduction.

## 2. Results and Discussion

### 2.1. De novo Reconstruction and Annotation of the Red Seaweed Grateloupia imbricata Transcriptome

The transcriptome of *G. imbricata* spans 11.6 Mb and was de novo assembled into 19,284 contigs.

Table 1 summarizes the assembly metrics and the sequence annotations; 10,866 contigs (hereafter referred to as transcripts) were annotated, and the remaining sequences (8418) correspond to unknown transcripts for which no homologs were identified in the current databases. The BUSCO results against the eukaryote database are according to the transcriptome size distribution, presenting mainly incomplete transcripts ([13]; Table 1). The average size of transcripts in our transcriptome are lower than the average size in other algae [14]; this size is an intrinsic problem of Ion Torrent technology and due to the fact that the BUSCO software transcriptome analysis is based in ORF analysis, the main result corresponds to fragmented (51.5% vs. 24.8% of complete). Our results show that only 23% of the studied orthologs appear as missing. Focusing on annotated contigs, 10,215 were coding sequences annotated using the NR protein database, and 651 were annotated using the NT database. Of the annotated sequences, 46% showed strong significance (e-values < 1 × 10^−15^) to their subjects, 25% were significant with e-values between 1 × 10^−14^ and 1 × 10^−5^, and the remainder were annotated based on e-values > 1 × 10^−5^. The greatest number of annotation top hits per subject species was provided by the red macroalga *C. crispus*, with ~43.7% of all subjects, followed by the unicellular red alga *G. sulphuraria*, with 7.1%. Of the entire set of sequences annotated as “coding”, 56% were assigned GO annotations and ECs and later were used to identify 99 metabolic pathways (retrieved online from the KEGG database, see Materials and Methods) that could be associated with the *G. imbricata* transcriptome. As for associations with known orthologs, the *G. imbricata* transcriptome could be classified into 2304 KOG clusters comprising 5394 transcripts annotated according to a distribution of e-values similar to that indicated for the annotation based on the NR/NT databases. See Table 1 and supplementary file 1 for additional information concerning all annotations.

### 2.2. Functional Profile of the Grateloupia imbricata Transcriptome

**Figure 1:**
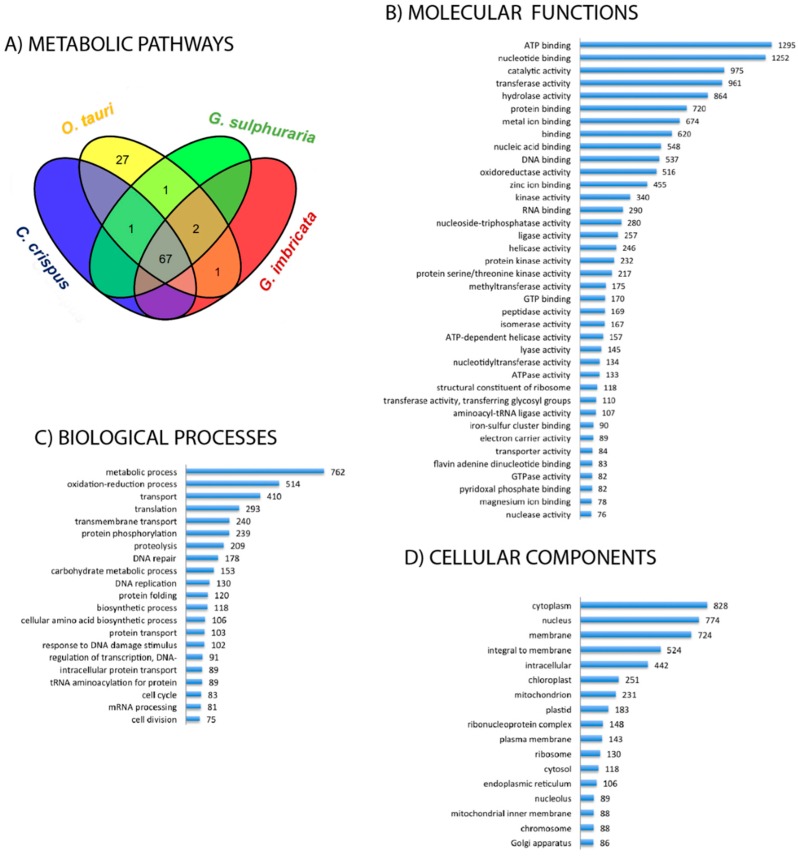
Metabolic pathways and inferred ontologies. (**A**) Venn diagram showing the metabolic pathways identified in *Grateloupia imbricata* and those of the three other algal taxa shared with *G. imbricata*. (**B**) GO terms at all levels in the GO DAG (direct acyclic graph) obtained per GO term “Molecular Function” after filtering terms with ≥75 annotated sequences. (**C**,**D**) The same information is presented for the GO terms “Biological Process” (C) and “Cellular Component” (D).

In the transcriptome of *G. imbricata*, the identification of ECs (i.e., referring here to the corresponding enzymes) that participate in 99 metabolic pathways allowed us to approximate a metabolic profile for this organism (supplementary file 1). To compare the metabolic profile of *G. imbricata* with that of other algae, a Venn diagram was created to determine which of the 99 metabolic pathways of *G. imbricata* are present in other taxa such as *C. crispus* and *G. sulphuraria* (both red algal taxa) or in *O. tauria* (green alga representative; Figure 1A). As approaches for transcript reconstruction rely on aligning reads to a reference genome, comparison to *C. crispus*, *G. sulphuraria* and *O. tauria* provided a solution for transcriptome reconstruction of *G. imbricata* in the absence of a reference genome.

According to this analysis, 91 of the 99 pathways are present in *C. crispus*, *G. sulphuraria* and *O. tauria*, suggesting that all four organisms have a common core of pathways for intermediary metabolism and the production of secondary metabolites. In this respect, the transcriptome of *G. imbricata* is particularly enriched in enzymes related to nucleotide metabolism (cellular source for energy and phosphate), information processing, energy production, the metabolism of carbohydrates, lipids, amino acids, cofactors, vitamins, and including enzymes involved in the chlorophyll-mediated biosynthesis of components related to both photosystems (I and II) and light-harvesting complexes. The Venn diagram also shows that *G. imbricata* generates transcripts for enzymes related to four pathways present in *C. crispus* and *G. sulphuraria* (degradation of chlorocyclohexane and chlorobenzene, biosynthesis of mucin-type O-glycan, retinol metabolism, and drug metabolism—other enzymes) and to one pathway of *C. crispus* (atrazine degradation). These pathways highlight certain compounds that may be of interest for studies of the processes related to the metabolism of terpenoids and polyketides, cofactors and vitamins, and xenobiotics and/or the biosynthesis of glycans and other secondary metabolites in red algae. The *G. imbricata* transcriptome also includes a transcript for an EC related to steroid degradation that also is related to transcripts encoding ECs of *G. sulphuraria*. To evaluate the *G. imbricata* transcriptome at the functional level, we carried out an analysis to determine the distribution of the abundance of the GO terms annotated to Molecular Function, Biological Process and Cellular Component. For subsequent analysis will only be taken into account those GO terms supported by at least 75 transcripts. Figure 1B shows the distribution of Molecular Function annotations, which demonstrates that the most prominent annotations correspond to transcripts involved in nucleotide binding or in enzymatic activities such as transferase, hydrolase, kinase, and oxidoreductase. As for Biological Processes, the analysis reveals the particular abundance of transcripts involved in metabolic activities, oxido-reduction processes, transport, translation, phosphorylation, proteolysis, DNA repair, metabolism of carbohydrates, and regulation (Figure 1C). Finally, the summary of Cellular Components suggests a predominance of transcripts-encoding proteins that function in the cytoplasm, nucleus, membranes, and plastids (Figure 1D). 

### 2.3. Metabolic Perspectives on the Growth, Development, and Reproduction of Grateloupia

Several benchmarks were considered when inferring metabolic information from our transcriptome evaluation of *G. imbricata*. First, *G. imbricata* and *C. crispus* share more metabolic pathways than other algal taxa, i.e., given that *C. crispus* was the top-hit species during our annotation of the *G. imbricata* transcriptome. Second, these two red seaweeds are the only red multicellular algae with commercial interest belonging to class Florideophyceae that have been sequenced so far [2,15] and this work. Hence, we selected *G. imbricata* transcripts with annotations related to sugar transport and uptake; ion/cation transport; floridoside and carrageenan synthesis; and the metabolism of polyamines, ethylene, and jasmonate as well as annotations related to stress pathways. Finally, annotations were interpreted and pathways were reconstructed based on information for *C. crispus* metabolism. 

**Figure 2:**
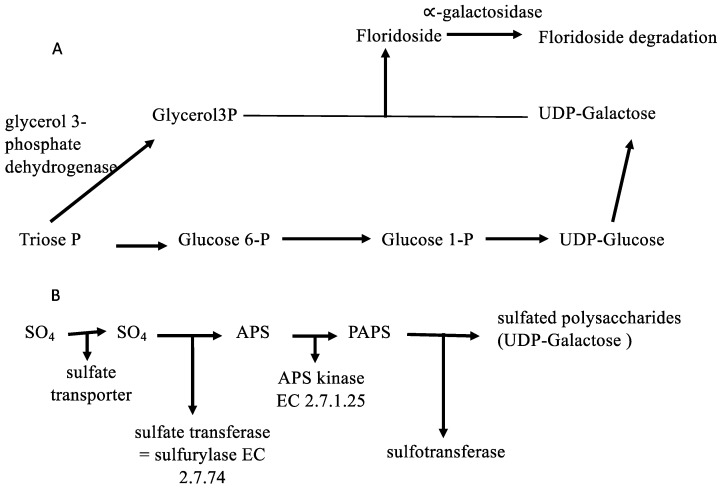
Biosynthetic pathways for the two principal polysaccharides of red algae. (**A**) Synthesis route of floridoside from UDP-galactose. (**B**) Sulfate assimilation and synthesis of sulfated polysaccharides.

Concerning carbon sources, a number of annotations could be obtained for the *G. imbricata* transcriptome regarding sugar transport and uptake, polysaccharide biogenesis, phosphorylation mechanisms, and transmembrane transport. These annotations were in fact anticipated based on the predicted protein trafficking patterns for *G. imbricata* during growth and development (supplementary file 2). Although the main products of carbon fixation in red seaweeds are sulfated polysaccharides (agar and carrageenan) and floridoside, as the most important low-molecular-weight carbohydrates, it is notable that the biosynthetic pathways for carrageenan and sugar transport have not been fully elucidated in red seaweeds [16]. Moreover, it has been assumed that UDP-galactose is the pivotal precursor for floridoside formation and polysaccharide biosynthesis and that polysaccharide biosynthesis occurs through the three sequential steps of linking sugar units, modifying hexose units, and activation via sulfation [17]. Likewise, our knowledge of sulfur assimilation and its contribution to the biosynthesis of sulfated polysaccharides is fairly limited in red algae. Although Robaina et al. [18,19] reported that *G. imbricata* thalli grows in glycerol-containing media and that oxygen evolution is altered when thalli are cultured in media with glycerol, little is known about the gene-encoding enzymes involved in these biosynthesis routes. Hence, an analysis of sequences involved in the uptake, sulfation, transport, and synthesis of sugars could serve as a starting point for studies of the factors that govern polysaccharide metabolism in seaweeds. Therefore, genes involved in polysaccharide biosynthesis were selected based on these aspects. We selected enzymes for the synthesis of two polysaccharides, namely floridoside and carrageenan, and specifically annotated glycerol 3-phosphate dehydrogenase (EC 1.2.12), which catalyzes the formation of glycerol 3-phosphate that condenses with UDP-galactose to produce floridoside phosphate [20] (Figure 2A). Moreover, α-galactosidase (EC 3.2.1.22), which mediates the degradation of floridoside, was also annotated [21] as were the gene-encoding enzymes for carrageenan biosynthesis, including galactosyl transferase, sulfotransferase, and galactose-6-sulfurylase (Figure 2B and supplementary file 2). Other enzymes such as sulfotransferase, also known as sulfurylase, are responsible for sulfation-mediated activation of substrates in eukaryotes [22], and several other sugar and polyol transporters are induced according to the available substrate(s); most transporters in fact can translocate more than one substrate [23]. Thus, knowledge of the pathways in which these genes participate during the growth and development of red algae can help decipher how sugars are transported and assembled and inform our understanding of the regulation of carrageenan synthesis. 

**Figure 3:**
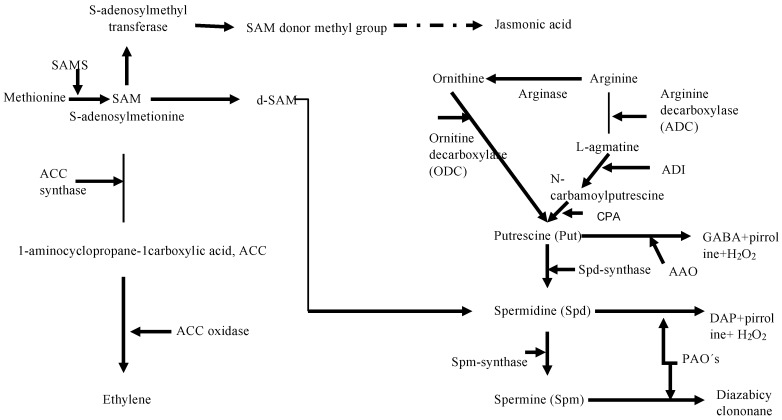
Biosynthetic pathway for polyamines and connections with the pathways for the biosynthesis of ethylene and jasmonate. S-adenosyl methionine synthase, SAMS; decarboxylated SAM, d-SAM; arginine decarboxylase, ADC; ornithine decarboxylase, ODC; 1-aminocyclopropane-1-carboxylic acid, ACC; spermidine/spermine synthase, Spd/Spm synthase; agmatine deimidase, ADI; N-carbamoylputrescine amidohydrolase, CPA.

Studies of in vitro carposporogenesis in *G. imbricata* have demonstrated that polyamines, ethylene, and methyl jasmonate act as elicitors of cystocarp development, spore release, and the timing of the development of reproductive structures; moreover, knowledge of the functional mechanisms of these elicitors has informed the search for candidate genes that govern reproduction in red algae [5,8,9,12]. Therefore, in our transcriptome analysis, we selected transcripts related to polyamine metabolism such as ornithine decarboxylase (ODC), SAMS, SAM decarboxylase, copper amine oxidase, spermidine/spermine synthase, putrescine aminopropyltransferase, agmatine deimidase, and N-carbamoylputrescine amidohydrolase (supplementary file 2). Polyamines play multiple physiological roles, including that of algal reproduction in different red seaweeds [4,6,7,8,9,10,24,25,26]. Once reproductive processes are initiated, polyamines are degraded by amine oxidases [6,7]. The synthesis of the obligate precursor for polyamine biosynthesis, namely putrescine, can proceed from the non-protein amino acid l-ornithine through the action of ODC (EC 4.1.1.17), and from arginine via the action of arginine decarboxylase [7] with intermediate deamination of agmatine. Molecular studies have shown that expression of *ODC* is an indicator of cystocarp maturation and sporulation in *G. imbricata* [9,10,11,12,26]. Although it seems the arginine decarboxylase pathway is generally not highly activated in algae [6,7], we found transcripts for the genes encoding the two enzymes carbamoylputrescine amidohydrolase and agmatine deimidase, which convert agmatine to putrescine (Figure 3). The identification of these two transcripts will facilitate studies of the balance of polyamines through cystocarp development and maturation.

Other groups of plant growth regulators meriting attention are those related to the metabolism of each of the jasmonates and ethylene, for which the main precursor is SAM, which is the substrate for 1-aminocyclopropane-1-carboxylate (ACC) synthase in the ethylene biosynthesis pathway and the main donor of methyl groups for jasmonate synthesis [27]. Figure 3 presents the biosynthesis pathways for polyamines, jasmonates and ethylene; those highlighted are key enzymes that are relevant to gene-level analyses of reproduction in seaweeds [9,26]. 

Methyl jasmonate induces both cystocarp maturation and sporulation [5]. In higher plants, jasmonic acid is synthesized by the oxygenation of linolenic acid via lipoxygenase, and a methyl group is then added to jasmonic acid, which yields methyl jasmonate; however, in seaweeds which perceive and respond to methyl jasmonate, the relevant biosynthetic enzymes have not been characterized. In this regard, we identified the nucleotide sequences related to methyl jasmonate metabolism described in higher plants. We identified transcripts encoding lipoxygenase and SAM methyltransferases, which catalyze methyl transfer and may also be associated with jasmonate biosynthesis in seaweeds (Figure 3 and supplementary file 2). The identification of these genes could promote a comprehensive understanding of algal physiology through gene overexpression and knockouts. We previously demonstrated that red seaweed thalli treated with methyl jasmonate exhibits induction of a dual gene response related to jasmonate signaling and the reproduction of thalli [11]. Other issues have remained unresolved, such as which gene networks are involved in reproduction and how the temporal alignment of particular events culminates in spore release. In this regard, comparative studies should be carried out to explain to what extent, if any, the gene network that governs cystocarp development is modified to generate mature reproductive structures. 

**Figure 4:**
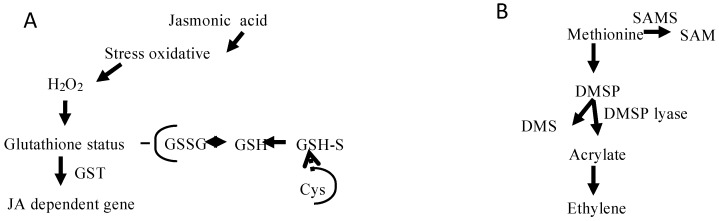
(**A**) Synthesis of jasmonate-related components; (**B**) alternative route for ethylene biosynthesis in algae. Glutathione sulfotransferase, GST; dimethylsulfopropionate lyase, DMSP lyase.

Also noteworthy is that our selection of transcripts includes those encoding jasmonate-responsive factors, which are not directly related to jasmonate biosynthesis but coevolved with signaling mechanisms and stress responses as demonstrated in higher plants [28]. The genes that take part in these jasmonate routes encode 1-deoxy-d-xylulose 5-phosphate synthase, 1-deoxy-d-xylulose 5-phosphate-reductosisomerase, farnesyl diphosphate synthase, geranyl diphosphate synthase, glutathione S-transferase, cytochrome P450, and tyrosine aminotransferase. Interestingly, genes that govern the synthesis of jasmonate derivatives may also be linked to oxidative stress because they lead to the generation of reactive oxygen species, including hydrogen peroxide, superoxide anions and hydroxyl free radicals [29]. Hence, our analysis reveals a summary of annotations for superoxide dismutases, catalases, ascorbate peroxidases, factors associated with protein folding and unfolding (e.g., heat shock protein WD 40), protein degradation factors (e.g., the 26S proteasome and ubiquitin), glutaredoxins, glutathione peroxidases, and/or for proteins involved in the early response to dehydration (supplementary file 2). Because genomic information concerning the genes involved in jasmonates biosynthesis is limited in seaweeds, our study will help researchers carry out analyses of the expression and function of these genes and take advantage of insights on alga reproduction. It is also worth stressing that although the role of jasmonates and the involvement of reactive oxygen species in *G. imbricata* reproduction were recently described [5], hitherto the genes have not been annotated for algae. Figure 4A shows the genes involved in jasmonic acid-responsive pathways, which presumably occur in seaweeds.

Finally, certain transcripts related to ethylene signaling and synthesis were selected according to their descriptions and annotations, as summarized in supplementary file 2. Concerning ethylene signaling, the selection includes different kinases such as the mitogen-activated protein kinase. Notably, it is known that perception of stimuli, such as gaseous hormones, activates different membrane receptors and signaling molecules [30,31]. It is important to highlight that environmental stimuli such as desiccation that occurs periodically in tidal pools and favors spore release [32] as well as water temperature and photoperiod that induce sporogenesis in red algae [33,34], mediate their effects via kinases and the downstream production of reactive oxygen species. Thus, these proteins may also mediate the cross-talk and signal transduction that occurs during algal development in a manner similar to that described by Schaller [35] in plants.

In relation to ethylene biosynthesis, four major enzymes mediate ethylene biosynthesis in marine organisms: SAMS (as previously noted), ACC synthase, ACC oxidase, and dimethyl sulfopropionate (DMSP) lyase [8,36]. Of these four enzymes, mRNAs for SAMS and ACC synthase were identified in the *G. imbricata* transcriptome, but we did not detect transcripts with annotations for ACC oxidase and DMSP lyase (Figure 3). In the red seaweed *Gelidium arbuscula*, Garcia-Jimenez et al. [37] demonstrated that ethylene is not generated as a consequence of the transformation of DMSP through DMSP lyase (refer to the model in Figure 4B). As DMSP is an osmoprotectant in red seaweeds [38], DMSP synthesis can occur through sulfonium compounds derived from methionine. Because the pathways that govern SAM, S-methylmethionine (SMM), and sulfur are poorly understood in algae [39], gene-encoding enzymes involved in sulfur pathways may help decipher the mechanism of DMS production in seaweed. 

Likewise, the ethylene receptor was also not annotated. Garcia-Jimenez and Robaina [9] reported that the structure of the *G. imbricata* ethylene receptor differs from those of other organisms, and indeed among higher plants there is great variation in the structure [30]. Despite defining the functions of genes encoding these enzymes in *G. imbricata*, additional gene expression information is necessary to clarify their roles in carposporogenesis in response to growth regulators. Therefore, these annotated sequences of the *G. imbricata* transcriptome can also be used as a potential starting point for further work on the pathways that guide reproductive processes in this red seaweed.

In conclusion, consideration of the genes identified in our transcriptome analysis with the subsequent annotation of gene functions may help identify deficits in our current knowledge of many aspects of seaweed reproduction and shed light on the genes that govern carposporogenesis. The BUSCO results support our transcriptome as a tool to understand the biology of G. imbricata. Unlike the mere annotation of a transcriptome, this work will allow future studies to focus on reproduction control. Biotechnology may also benefit from these data because certain aspects of reproduction, such as reproductive stages, may inform technological innovation—most prominently by controlling the synthesis of cell-wall polysaccharides.

## 3. Materials and Methods

### 3.1. Sampling

*Grateloupia imbricata* (Florideophyceae) was sampled as whole individuals without visible epiphytes from the northeastern coast of Gran Canaria island (Canary Islands, Spain). At 1 h post-collection, thalli were selected, brushed, removed of associated biota, and acclimatized for 1 day in sterilized seawater. Additionally, thalli were frozen in liquid nitrogen before storage at −80 °C. 

### 3.2. RNA Extraction and Poly(A)-RNA Enrichment

RNA isolation was carried out on six different days and with four independent replicates for each one. Then, 12 of the 24 samples were chosen randomly and pooled for assays. The pooling samples enabled to obtain a more reliable transcriptome and to overcome possible inter-individual variations of gene expression. The pooling samples enabled to obtain a more complete transcriptome and to overcome possible inter-individual variations of gene expression. Total RNA was isolated from thalli (100 mg) using a NucleoSpin RNA plant kit (Machery Nagel, Neumann, Germany). The quality of the total RNA was checked using 2% agarose denaturing gel electrophoresis by inspection of the integrity of 28S and 18S rRNA bands. Then, samples were further purified with a RNA clean-up XS kit (Machery Nagel). As assessed with the Bioanalyzer System of Agilent Technologies (Santa Clara, CA, USA), samples with a 28S/18S rRNA ratio between 1.7–2.0 and a RIN (RNA Integrity Number) 9–10 were selected for poly(A)-RNA enrichment in two successive rounds using a Dynabeads mRNA direct microkit (Life Technologies, Carlsbad, CA, USA) following the manufacturer’s instructions with some modifications. In short, 8 μg total RNA in a microtube was diluted to 150 μL with nuclease-free water and heated at 70 °C for 2 min. After the addition of an equivalent volume of lysis/binding buffer from the kit, samples were mixed with 30 µl of oligo-d(T) magnetic beads (10 times) and incubated for 5 min at room temperature. The resultant enriched poly(A)-RNA samples were resuspended in 50 µL elution buffer from the kit, divided into three aliquots, and stored at −80 °C. The concentration of poly(A)-RNA was measured using a Qubit 2.0 fluorometer (Qubit RNA assay kit, Invitrogen, Paisley, UK) as well as the Agilent Bioanalyzer System.

### 3.3. RNA Library Construction and Transcriptome Sequencing

Two cDNA libraries (1 and 2) were constructed and sequenced using a PGM Ion Torrent platform (Life Technologies) for next-generation sequencing. Both libraries were sequenced independently to increase the sequencing deep and overcome the Ion Torrent limitations; in addition, both libraries can be used as technical replicates. Poly (A)-RNA (300 ng) was used as starting material. The first step consisted of a partial digestion with RNase III at 37 °C for 3 min. The length, size distribution, and concentration of the fragmented RNA was assessed using the Agilent Bioanalyzer with an RNA 6000 Pico kit (Agilent Technologies). Then, 50 ng of material was used in the AB Library Builder system (Applied Biosystems) with the Ion Total RNA-Seq kit (Life Technologies) to automatically construct each library. Double-stranded cDNA, obtained by consecutive reverse transcription and PCR amplification, was also analyzed with the Agilent Bioanalyzer using a high-sensitivity DNA kit (Agilent Technologies). Samples were then serially diluted such that the smallest concentration was 23 pM and subjected to emulsion PCR and enrichment using the OneTouch system (Ion OneTouch 200 Template v2DL kit, Life Technologies). Finally, each sample was loaded on a single Ion 316 chip and sequenced in two consecutive runs using the same initialization procedure on the PGM platform. The sequencing data for both cDNA libraries was outputted as two fastq files, one for library 1, with 3,431,580 raw reads, and the other for library 2, with 2,946,415 raw reads, for a total of 6,377,995 raw reads. The average and maximum sequence lengths were 250 and 308 bp, respectively. 

### 3.4. Preprocessing, De Novo Assembly, and Annotations

The quality of the two fastq files was assessed using FastQC tools (Version 0.11.8, Babraham Institute, Cambridge, UK). Prinseq [40] was used to pre-process both fastq files, removing low-quality sequences (phred quality score <15) and artifacts. The pre-processing step yielded 6,345,850 clean reads, which were de novo assembled into 19,284 contigs using the CLC-bio assembler integrated in the suite clc-assembly-cell-4.2.0 (CLC assembly-cell 5.0.0, Institut Pasteur, C3BI, Paris. France) with default parameters. The BLAST pipeline of GPRO [41] was used to annotate the G. imbricata transcriptome against the non-redundant refseq databases of peptides (NR) and nucleotides (NT) at NCBI using the program BlastX [42]. This pipeline includes annotation of Gene Ontology (GO) terms [43] and enzyme commission (EC) numbers. Information about metabolic pathway maps was also retrieved via the web from the KEGG database [44] based on EC numbers. The BLAST pipeline was also used to annotate orthologs from the G. imbricata transcriptome in the Eukaryotic Orthologous Groups (KOG) database of NCBI [45]. Contigs annotated as coding were translated to peptide sequences using Transeq from EMBOSS implemented online at EMBL-EBI (EMBL-EBI 2018, Wellcome Genome Campus, Hinxton, Cambridgeshire, UK), whereas contigs for which no significant protein hit was detected (unclassified sequences) were translated to putative peptides using the OrfPredictor server (Web Server Issue W677-W680, Youngstown State University, Youngstown, OH, USA). 

### 3.5. Data Mining and Comparative Analyses

To compare the metabolic profile of *G. imbricata* with that of other algae, the KEGG database website was browsed to obtain information on the metabolic pathways of the red multicellular alga *Chondrus crispus*, the unicellular red alga *Galdieria sulphuraria*, and the green alga *Ostreococcus tauri*. To complement the information provided by KEGG, different searches of the Entrez site at NCBI with the Ensembl Biomart platform [46] were performed to retrieve all protein annotations available in GenBank for the aforementioned three algae. Then a summary of ECs and metabolic pathways was obtained for each taxon using methods identical to those applied for the *G. imbricata* transcriptome. Subsequently, GPRO software (GPRO suite by Biotechvana) was used to perform statistical analyses, for which Venn diagrams were generated based on the metabolic pathways of *G. imbricata* and those of the aforementioned taxa. 

### 3.6. Data Availability

Raw data were deposited at the NCBI Sequence Read Archive (SRA) with BioProject record PRJNA309128 and BioSample record SAMN04420758. Contig sequences, protein predictions of at least 100 amino acid residues in size, and all annotations performed for the *G. imbricata* transcriptome are presented in the supplementary material (Supplementary file 1).

## Figures and Tables

**Table 1:** Assembly metrics and Annotations for the *Grateloupia imbricata* transcriptome.

Contigs/Transcripts	19,284	
Total transcriptome size	11,681,373	
Longest transcript (nt)	7289	
Shortest transcript (nt)	200	
Number of transcripts < 1K	16,664 (86.3%)	
Number of transcripts > 1K	2640 (13.7%)	
Mean transcript size	606	
Median transcript size	400	
N50 (nt)	734	
L50 (nt)	4176	
%A	25.72	
%C	24.99	
%G	24.45	
%T	24.84	
%Ns	0	
Average coverage	42.28	
Databases/Systems	NR annotations	Sequences
NR/NT Gene Identifiers (GIs)	8326	10,866
Gene Ontology (GO) terms	3323	5686
Enzyme Codes (ECs)	637	2087
Metabolic Path Maps	88	2031
Orthologs (KOG) Clusters	2304	5394
BUSCO Parameters		
Complete	Total	25.5%
	single-copy	24.8%
	duplicated	0.7%
Fragmented	51.5%	
Missing	23.0%	
Metrics were Inferred using the script assemblathon_stats.pl available (version 3.0. Korf Lab. Genome Center, UC Davis. USA)		

## Supplementary Materials

Supplementary materials can be found at http://www.mdpi.com/1660-3397/16/12/490/s1. Supplementary file 1 File SP11: fasta file containing the full set of sequences of transcripts constituting the transcriptome of *G. imbricata*. File SP12: fasta file with the protein sequences of coding transcripts. File SP13: Excel file containing five subfiles: (1) automatic annotation file of the *G. imbricata* transcriptome based on NR/NT databases plus annotation of GO terms and ECs; (2) distribution of annotations per subject species; (3) annotation file of KOGs; (4) summary of all metabolic pathways elucidated for *G. imbricata*; (5) assignation of annotated ECs and transcripts to distinct metabolic pathways. Supplementary file 2 Selection of transcripts with GO annotations related to: polyamine metabolism; sugar transport and uptake, and ion/cation transport; floridoside and carrageenan synthesis; jasmonate metabolism; stress; and ethylene metabolism.
